# Long-term efficacy and safety of ab externo canaloplasty in the Polish Caucasian population with open-angle glaucoma: A 3-year retrospective study

**DOI:** 10.1371/journal.pone.0312236

**Published:** 2024-10-16

**Authors:** Joanna Konopińska, Kinga Gołaszewska, Emil Saeed

**Affiliations:** Department of Ophthalmology, Medical University of Białystok, Białystok, Poland; University of Missouri-Columbia, UNITED STATES OF AMERICA

## Abstract

This study aimed to assess the effectiveness and safety of ab externo canaloplasty over a 3-year follow-up period in Polish Caucasian patients with glaucoma. This retrospective study of a cases series was conducted at the Department of Ophthalmology Medical University of Bialystok, Poland. Primary outcome measures were intraocular pressure and medication burden. Secondary outcome measures were best corrected visual acuity, retinal nerve fiber layer thickness, visual field test results, endothelial cell density, intraoperative and postoperative complications, and additional glaucoma surgery. The study group consisted of 160 eyes diagnosed with early to-moderate primary open-angle glaucoma and treated with ab externo canaloplasty from 2014 to 2020. The criteria for qualified and complete success were intraocular pressure < 18 mmHg or ≥ 20% reduction in intraocular pressure from baseline with or without antiglaucoma medications, respectively. After surgery, the mean intraocular pressure decreased from 19.23 ± 4.33 to 14.52 ± 3.79 mmHg, which constituted a 36.7 ± 7.8% reduction compared to the baseline value. The number of medications decreased by two at the end of the study period (from 2.69 ± 1.05 to 0.32 ± 0.75). Complete and qualified success were achieved in 58.7% and 68.7% of the patients, respectively, at the end of follow-up. An incomplete cannulation of Schlemm’s canal was the most common intraoperative complication, occurring in 32 eyes (20%). The most frequent postoperative complications were hyphema, Descemet’s membrane detachment, and a transient increase in intraocular pressure. Additional surgical procedure was required in seven cases (4.4%). In 16 patients (10%), medical treatment was re-administered. Overall, our findings suggest that ab externo canaloplasty is a good option for Polish Caucasian patients with primary open-angle glaucoma. It reduces intraocular pressure and has a low postoperative complication rate. Additional glaucoma surgery or re-administration of medications is required if the target intraocular pressure is unsatisfactory.

## Introduction

Glaucoma is one of the most common causes of irreversible blindness worldwide, and a reduction in intraocular pressure (IOP) is the only proven preventive factor. When pharmacological or laser treatments are ineffective, glaucoma surgery should be considered to prevent further damage to the optic nerve. The estimated global prevalence of open-angle glaucoma (OAG) is 3.5% in individuals aged 40–80 years [1, 2]. Primary OAG (POAG) is the most common type of glaucoma and exhibits significant diversity in its distribution according to ethnicity. African Americans have the highest prevalence of POAG (approximately 5.6%) and are more prone to rapid progression towards blindness compared to other ethnic groups. Latinos are ranked second in terms of the POAG prevalence (4.7%), followed by Asians and non-Latino Whites, with POAG prevalence rates of 2.4% and 1.7%, respectively [1].

Differences in the effectiveness and safety of various glaucoma treatment modalities, particularly among ethnic groups, have been described in the literature [3]. Okeke found that the outcomes of trabectome use were slightly better for Spanish than for non-Spanish White, African-American, or Asian patients [4]. In another study, Gallardo et al. reported that the first generation of iStent reduced the IOP from 16.5 mmHg to 12.9 mmHg (by 3.6 mmHg) and that medication reduced it by 1.4 (from 2.3 to 0.9) in Spanish patients [5]. These results are superior to those of a similar study of non-Latino patients, where the IOP decreased from 19.1 to 15.5 mmHg (by 2.6 mmHg) and medications made it drop by 0.6 (from 1.2 to 0.6) at 12 months [6].

Chen et al. reported that the diversity in susceptibility to glaucoma treatment stems from differences in both the anatomy of Schlemm’s canal and the structure of the trabecular meshwork (TM), which may vary depending on the population [7]. Africans are reported to have a lower TM height, which among other factors may reduce the rate of aqueous humor (AH) outflow [8–13]. Moreover, the success of trabeculectomy is less likely in Black individuals than in White individuals because of excessive fibrosis in the subconjunctival space [7–9], which may result from differences in the cellular profile of the conjunctiva. Specifically, there are more fibroblasts and macrophages in the conjunctiva of Black individuals, which may cause surgical failure [8]. In addition, patients with POAG have fewer cells in the TM than do healthy individuals, and this effect is much more evident in the TM of African-American people [10]. Similarly, individuals of Latin descent, especially those of African ancestry, have unique anatomical and physiological characteristics, such as scleral tensile strain, a longer axial length, thin corneas, and corneal hysteresis, all of which may play a role in the risk of OAG progression in these populations [14]. In Asian populations, additional factors such as low diastolic pressure, low diastolic perfusion pressure, and low mean ocular perfusion pressure have recently been reported as additional risk factors for POAG [15]. Determining the best approach for treating certain populations with OAG is a public health challenge.

According to the recommendations of the European Glaucoma Society [16], trabeculectomy is the gold standard for the surgical treatment of glaucoma. It is effective in reducing the IOP by 47–65% compared to baseline [17]. Its hypotensive effect is achieved by creating an additional pathway for the outflow of AH into the subconjunctival space, which ensures high efficacy. However, trabeculectomy is also associated with a high-risk profile of vision-threatening complications; therefore, it is commonly used in advanced stages of the disease. In addition, its effectiveness decreases over time within the postoperative period.

Ab externo canaloplasty is a hybrid procedure that combines viscocanalostomy and nonpenetrating deep sclerectomy [18]. The classical surgical technique is based on the creation of a trabeculodescemetic membrane (TDM), an intrascleral-scleral lake, and intubation of Schlemm’s canal [19]. In the case of canaloplasty, the mechanism of hypotensive action is complex since it operates through several mechanisms. First, a suture incorporated into the Schlemm’s canal enhances the permeability of the TM, SC, and juxtacanalicular tissue. This improves the outflow of AH through the conventional pathway. Another probable mechanism for lowering IOP is drainage of the AH through the TDM into the intrascleral decompression space, which serves as a reservoir, thus enabling prolonged absorption of the AH and its trans-scleral and subconjunctival outflow [20–23]. Canaloplasty potentially affects all levels of conventional AH outflow, both distal and proximal.

Based on previous literature, we speculated that the efficacy of canaloplasty may vary depending on the population owing to anatomical differences in the structure of the eyeball. The aim of our study was to assess the effectiveness and safety of ab externo canaloplasty in a Polish Caucasian population over a 3-year follow-up period. According to our investigation, the procedure is safe and effective. The potential implications of this study are that it could contribute to a better understanding and treatment of glaucoma in Polish Caucasian population. This approach may potentially affect practices, policies, and future research.

## Materials and methods

This retrospective study of a case series was conducted at the Ophthalmology Department Medical University of Białystok, Poland, and included 160 eyes diagnosed with early-to-moderate primary open-angle glaucoma (POAG) that underwent ab externo canaloplasty from 2014 to 2020. The patient population comprised 93 women and 67 men aged 67.35 ± 10.54 years on average. Data from a 36-month follow-up period are included. This study was performed in line with the principles of the Declaration of Helsinki. The protocol was approved by the Local Bioethics Committee at the Medical University of Bialystok (approval number: R-I-002/444/2014. Informed written consent was obtained from all patients before the study.

The database was accessed and the data were collected between 10^th^ and 15^th^ January 2024. Patient data were fully anonymized for analysis.

Patients with POAG, in whom the progression of visual field defects had been documented despite maximally tolerated hypotensive treatment, were qualified to undergo elective glaucoma procedure. The other inclusion criteria were as follows: significant diurnal fluctuations in IOP, lack of patient compliance with glaucoma therapy, and allergy to topical medications. The exclusion criteria comprised refusal to undergo surgery, previous eye surgery and laser procedures except for phacoemulsification, narrow or angle closure glaucoma, secondary, post-inflammatory, or post-traumatic glaucoma, chronic corneal disease and corneal opacity preventing gonioscopy examination, optic nerve disorders other than glaucomatous optic neuropathy, advanced macular degenerative disease, active inflammatory process, pregnancy, and systemic steroid therapy. Eyes with medicated IOP of 18 mmHg or lower were qualified for surgery due to medication intolerance. A single eye per patient was included in the research and where both eyes qualified, the first eye treated was selected for analysis.

### Pre-surgery medical procedure

The primary outcome measures were IOP and medication burden. Secondary outcome measures included: best-corrected visual acuity (BCVA) according to Snellen notification, retinal nerve fiber layer thickness in optical coherence tomography (OCT, Spectralis, Heidelberg Engineering, Heidelberg, Germany), visual field test results (Humphrey, SITA Standard 30–2), endothelial cell density (ECD), intraoperative and postoperative complications, and additional glaucoma surgery. The patients were interviewed in detail regarding their medications, allergies, and underlying health conditions.

### Surgical technique

The detailed surgical technique has been previously described [22]. Briefly, after dissecting the superficial and deep flaps of the sclera and creating a TDM, a catheter with an illuminated tip is inserted into Schlemm’s canal (iTrack 250A, iScience Interventional, Menlo, Park, CA, USA), allowing it to pass through the entire canal. As the microcannula is advanced into the canal, Hialon GV (Johnson & Johnson Vision, CA, USA) is injected to facilitate dilation and maintain patency. When the tip of the catheter reaches the opposite opening of the Schlemm’s canal, a 10–0 polypropylene tension suture is attached (Prolene; Ethicon, Inc.). The suture is then inserted into Schlemm’s canal to create tension on the trabecular meshwork. The superficial scleral flap is tightly sutured using nonabsorbable sutures. The conjunctival incision is closed using absorbable sutures.

### Post-surgery protocol

After the surgery, the patient was administered levofloxacin eye drops three times a day for 7 days and dexamethasone four times a day for 1 month, which was subsequently gradually tapered off by one drop per week. All glaucoma medications were discontinued on the day of the surgery. If the surgery did not yield the expected results, medications were reintroduced according to the recommendations of the European Glaucoma Society [16]. Postoperative follow-up visits were scheduled at 1 day, 2 weeks, and 1, 3, 6, 12, 24, and 36 months after surgical intervention. Within the follow-up period, in addition to measuring the IOP and BCVA, the anterior chamber and fundus of the eye were examined. The postoperative course was evaluated considering complications and the number of glaucoma medications. Hypotony was defined as an IOP ≤ 6 mmHg. Visual field testing and OCT was performed at 6, 12, 24, and 36 months after surgery. Surgical success was defined as the percentage share of patients with an IOP ≤ 18 mmHg or a decrease in the IOP ≥ 20% at the end of the follow-up period (complete: without medications, qualified: with or without medications).

Statistical analyses were performed using R version 3.5.1 (Vienna, Austria). The variables are presented using descriptive statistics. Data are shown as mean ± standard deviation. The normality of the distribution of quantitative variables was assessed using the Shapiro–Wilk test, data skewness and kurtosis indices, and visual assessment of histograms. Equality of variances was checked using Bartlett’s test. The comparative analysis of the results between the beginning and the end of the study was performed using Student’s *t*-test for dependent measurements. The mean difference (MD) was also calculated with the 95% confidence level. Additionally, the cumulative incidences of complete and satisfactory success were calculated using Kaplan–Meier survival analysis. Missing values were omitted from the analysis of individual variables. The significance level used was α = 0.05, all tests were two-sided.

## Results

### Demographics and baseline parameters

The **s**tudy group consisted of 160 patients (58.1% female) with an average age of 67.35 ± 10.54 years. The baseline characteristics are summarized in Table 1.

**Table 1:** Demographics and baseline characteristics of the study participants.

Variable	n (%)	Mean ± SD	Median (Q1; Q3)
N	160 (100.0)		
Sex, female	93 (58.1)		
Age, years		67.35 ± 10.54	68.00 (60.75; 75.25)
BCVA		0.73 ± 0.29	0.80 (0.55; 1.00)
IOP, mmHg		19.23 ± 4.33	19.70 (16.75; 25.00)
IOP < 18	16 (10)		
IOP ≥ 18 and ≤ 21, mmHg	60 (37.5)		
IOP > 21, mmHg	84 (52.5)		
No. of medications		2.69 ± 1.05	3.00 (2.00; 3.00)
ECD, cells/mm^2^		2274.12 ± 335.37	2240.00 (2107.00; 2498.50)

Notes: SD, standard deviation; Q1, 1^st^ quartile; Q3, 3^rd^ quartile; BCVA, best corrected visual acuity; IOP, intraocular pressure; ECD, endothelial cell density; SD, standard deviation

Data are presented as n (%) for nominal variables and mean ± SD and median (Q1; Q3) for numerical variables.

### Variation in the parameters over time

**Fig 1:**
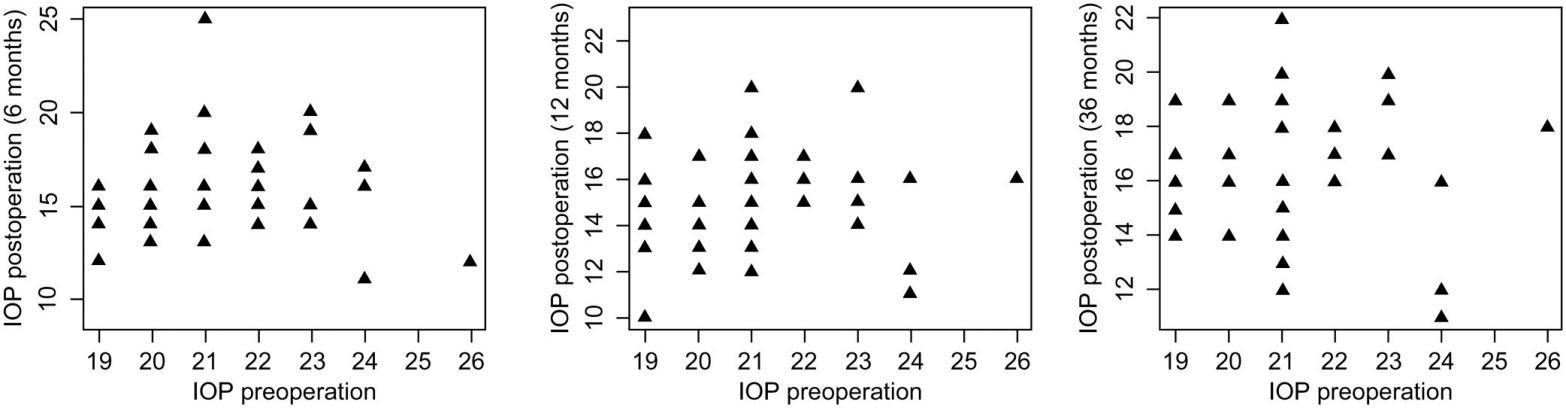
Scatterplots of IOP at different time points. IOP, intraocular pressure.

The variation in parameters at 180 days, 360 days, and 3 years versus baseline values was examined. The IOP level decreased significantly at each time point. After 180 days, it had decreased by averagely 4.75 mmHg (MD = -4.75, CI_95_ [-7.50; -3.50], p < 0.001); after 360 days, it had decreased by averagely 4.00 mmHg (MD = -4.00, CI_95_ [-5.00; -2.00], p = 0.025); and after 3 years, it had decreased by averagely 3.20 mmHg (MD = -3.20, CI_95_ [-3.50; -1.85], p < 0.001) (Fig 1). A significant decrease was found in the number of medications over time (MD = -3.00, CI_95_ [-3.00; -2.00], p < 0.001 at 180 days; MD = -3.00, CI_95_ [-3.00; -2.50], p < 0.001 at 360 days and 3 years). Additionally, a significant drop in ECD levels was observed after 180 days (MD = -337.71, CI_95_ [-593.53; -81.90], p = 0.014). No significant difference was observed in BCVA, RNFV, AV, and MD at any point (p > 0.05; Table 2).

**Table 2:** Variation in the parameters after 180 days, 360 days, and 3 years.

Time	n	Mean ± SD	Median (Q1; Q3)	MD (95% CI)	*p*
**BCVA**					
Baseline	160	0.73 ± 0.29	0.80 (0.55; 1.00)		
180 days	157	0.77 ± 0.28	0.90 (0.60; 1.00)	0.15 (-0.05; 0.25)	0.107
360 days	150	0.82 ± 0.23	0.90 (0.80; 1.00)	0.10 (-0.05; 0.35)	0.091
3 years	150	0.76 ± 0.34	1.00 (0.55; 1.00)	0.10 (-0.10; 0.15)	0.452
**IOP, mmHg**					
Baseline	160	19.23 ± 4.33	19.70 (16.75; 22.00)		
180 days	157	15.39 ± 2.00	15.00 (14.25; 17.00)	-4.75 (-7.50; -3.50)	**< 0.001**
360 days	150	15.00 ± 9.13	16.00 (15.00; 16.00)	-4.00 (-5.00; -2.00)	**0.025**
3 years	150	14.52 ± 3.79	15.00 (13.50; 17.65)	-3.20 (-3.50; -1.85)	**< 0.001**
**No. of medications**					
Baseline	160	2.69 ± 1.05	3.00 (2.00; 3.00)		
180 days	157	0.05 ± 0.29	0.00 (0.00; 0.00)	-3.00 (-3.00; -2.00)	**< 0.001**
360 days	150	0.08 ± 0.42	0.00 (0.00; 0.00)	-3.00 (-3.00; -2.50)	**< 0.001**
3 years	150	0.32 ± 0.75	0.00 (0.00; 0.00)	-3.00 (-3.00; -2.50)	**< 0.001**
**ECD, cells/mm** ^**2**^					
Baseline	160	2274.12 ± 335.37	2240.00 (2107.00; 2498.50)		
180 days	157	1989.67 ± 584.41	2008.00 (1769.50; 2282.00)	-337.71 (-593.53; -81.90)	**0.014**
360 days	150	2134.86 ± 739.65	2270.00 (1751.50; 2532.00)	-245.43 (-745.06; 254.20)	0.275
3 years	150	1982.68 ± 508.90	1969.00 (1759.00; 2398.00)	-23.33 (-495.73; 449.06)	0.851
**RNFL, μm**					
Baseline	160	68.59 ± 17.81	71.00 (55.00; 82.00)		
180 days	157	70.44 ± 21.09	66.00 (59.00; 83.00)	1.00 (-12.69; 14.69)	0.849
360 days	150	59.00 ± 14.93	53.00 (50.50; 64.50)	-0.67 (-12.14; 10.81)	0.826
3 years	150	73.12 ± 19.99	68.50 (57.25; 88.50)	-4.28 (-11.00; 2.45)	0.197
**MD, dB**					
Baseline	160	-8.71 ± 9.51	-6.92 (-13.24; -3.53)		
180 days	157	-9.55 ± 8.28	-8.22 (-13.87; -2.63)	-0.17 (-1.94; 6.82)	0.389
360 days	150	-10.02 ± 7.30	-7.92 (-12.86; -4.82)	1.72 (-2.78; 2.63)	0.625
3 years	150	-7.05 ± 6.89	-4.06 (-7.88; -2.86)	2.79 (-1.26; 3.15)	0.240

Notes: SD, standard deviation; Q1, 1^st^ quartile; Q3, 3^rd^ quartile; BCVA, best corrected visual acuity; IOP, intraocular pressure; MD, mean deviation; RNFL, retinal nerve fiber layer; CI, confidence interval

Time periods were compared using the paired *t*-test (CD, RNFL AV) or Wilcoxon test (BCVA, IOP, no medications, MD), as appropriate.

### Surgical success

The number of successful surgical cases and their prevalence within the relevant groups are presented in Table 3.

**Table 3:** Surgical success with and without medications.

Variable	180 days	360 days	3 years
**IOP drop ≥ 20%**			
All	114 (71.2)	110 (68.7)	110 (68.7)
With medications	24 (15.0)	16 (10.0)	16 (10.0)
Without medications	90 (56.2)	94 (58.7)	96 (58.7)

Notes: IOP, intraocular pressure.

Data are presented as n (% of all cases, with and without surgical success). The breakdown of medication use is based on the follow-up period.

### Complications

The most common intraoperative complications were the failure of the catheter to pass through Schlemm’s canal and the incorrect passage of the catheter, which occurred in 32 patients (20% of the cases). The eyes of the patients in whom the complete Schlemm’s canal cannulation and insertion of the micro-cannula at 360° had failed—had undergone deep sclerectomy and were excluded from the analysis; however, the patients continued to be monitored. The eyes of four patients (2.5%) who experienced TDM perforation and conversion to trabeculectomy during the course of surgery were excluded from the analysis as well. The most common postoperative complication was the presence of hyphema in the anterior chamber, occurring in 88 (55%) of cases. In all cases, the condition was transient and subsided spontaneously within 7–28 days. The postoperative IOP spike was observed in 24 (15%) of patients undergoing canaloplasty. Among them, 92% had a transiently elevated IOP, and eight (5%) showed a persistently high IOP, which was regarded as surgical failure. These patients received additional glaucoma surgery (Table 4).

**Table 4:** Intra—And post-surgery complications following canaloplasty.

Variable	Canaloplasty
**Intraoperative and early postoperative complications**	
Rupture of the trabeculo-descemetic membrane	4 (2.5)
Schlemm’s canal cannulation not possible	32 (20)
Microhyphema	88 (55)
Elevated IOP	24 (15)
Iritis	3 (2)
Descemet’s membrane detachment	3 (2)
**Late postoperative complications**	
Elevated IOP	8 (5)
Macular edema	0 (0.0)
Blurry vision or visual disturbance	2 (4.3)
Suture extrusion through the trabecular meshwork	1 (0.6)
Hypotony (IOP of 6 mmHg with a shallow anterior chamber)	N/A
Blebs at 36 months	4 (2.5)
Cataract	8 (5)

Notes: IOP, intraocular pressure; N/A, not applicable.

Data are presented as n (%). The groups were compared using the Pearson chi-squared^1^ test or Fisher’s exact test.

### Additional procedures

Post-surgical laser goniopuncture was performed in 39 patients (24.4%) due to an uncontrolled the postoperative IOP. The mean time from surgery to laser goniopuncture was 13 days (range: 6–21 days). In 32 cases, there was a substantial drop in IOP to the desired level. If the IOP did not reach the target pressure, IOP-lowering medications were readministered according to the recommendations of the European Glaucoma Society. In seven cases, additional surgical procedures were required, and classical trabeculectomy was performed. Among them, three patients underwent surgery within 6 weeks after the first surgery due to severe IOP spikes (>30 mmHg), and four other cases underwent surgery in the second year of follow-up. These patients were excluded from the analysis (their previous results were not withdrawn from the database). One patient was stitched up with an additional conjunctival suture due to separation of the conjunctival wound. Three patients were lost during follow-up.

## Discussion

Our results confirmed that canaloplasty effectively lowered IOP in Caucasian patients with POAG throughout the 36-month follow-up period. The mean IOP decreased from 19.23 ± 4.33 at baseline to 14.52 ± 3.79 mmHg (by 37.2%) at the end of the follow-up period. Throughout the study period, the number of glaucoma medications significantly dropped from 2.69 ± 1.05 to 0.32 ± 0.75 (preoperatively and 36 months postop., respectively). Our findings are consistent with those of previously published studies on Caucasian populations, which showed a promising reduction in IOP with few postoperative complications, as well as a decrease in medication burden [24, 25].

Bull et al. [24] conducted a prospective, multicenter study of Caucasian patients that aimed to compare ab externo canaloplasty (study group) to ab externo canaloplasty with phacoemulsification (control group), showing IOP reductions of 33.4% and 43.2%, respectively. In eyes undergoing canaloplasty alone, the IOP was reduced to 15.1 ± 3.1 mmHg 3 years after surgery. In the eyes that underwent the combined surgery, the IOP decreased to 13.8 ± 3.2 mmHg 3 years following the surgery, which emphasized the role of cataract removal in lowering the IOP in patients with OAG. Similar to our study, the most common early postoperative complications were microhyphema (blood level < 1 mm in the anterior chamber), hyphema (blood level > 1 mm in the anterior chamber), an IOP spike, and Descemet’s membrane detachment. Cases of hypotony or shallowing of the anterior chamber have not been reported.

A greater reduction in IOP was achieved in a study conducted by Grieshaber et al. [26], who examined 60 eyes of Black patients who had undergone ab externo canaloplasty. At the end of the 3-year follow-up period, the IOP decreased from the baseline value of 45.5 mmHg to 13.5 mmHg, accounting for a 65.8% decrease from baseline, and the number of medications decreased by two at the end of the follow-up period. In 98% of patients, the surgery was performed as a primary surgical intervention without any prior conservative treatment. The complete surgical success rate was much higher than in the abovementioned studies [24] and accounted to 77.5% and the qualified success rate was 81.6% (for the IOP≤21 mmHg). However, this phenomenon was previously described [27]: the eyes with a baseline IOP of >30 mmHg demonstrate more pronounced reduction of IOP (by more than 50%) compared to that of eyes with a baseline IOP of <20 mmHg, which experienced only a 30% reduction.

Liang et al. [28] conducted a study on the effectiveness of ab externo canaloplasty in Asian patients with POAG. The authors compared three modifications of classical techniques: canaloplasty with 5/0 suture, 6/0 suture, and microcatheter. The mean follow-up period was 14.8 ± 3 months. These authors achieved slightly better results than those of our study. The mean IOP decreased from 26.2 ± 2.7 to 14.5 ± 2.7 mmHg postoperatively, which constituted a 42.4% reduction from baseline (in comparison to 36.1% in the current study). The success rate in a criterion IOP < 18 mmHg and 20% IOP drop was achieved in 86.1% (qualified) and 61.1% (complete) (in comparison to our study at 12 months: 68.7% [qualified] and 58.7% [complete]). The most common complications included hyphema (30.6%), an IOP spike > 25 mmHg (8.3%), and peripheral synechia to the trabecular-Descemet’s membrane (2.7%). Another study of Asian patients was conducted by Fujita et al [29]. In the study group, mean preoperative IOP was 23.4 ± 5.5 mmHg and decreased postoperatively to 15.0 ± 4.1 mm Hg at 12 months, which constituted a 36% drop from baseline. The mean number of antiglaucoma medications was 2.8 ± 0.6 before canaloplasty and decreased to 1.2 ± 0.8 at 12 months after the surgery (p < 0.01). The most frequent postoperative complication was mild hyphema (45.5%), which resolved within 2 weeks after the surgery.

Gallardo et al. [30] conducted a study on canaloplasty ab interno and trabeculotomy in a Hispanic population. The unmedicated preoperative IOP was 22.8 mmHg and dropped to 14.9 mmHg postoperatively (by 34.6%). Further, the average number of medications dropped from 2.0 to 0.15 postoperatively. The most common complications were an IOP increase ≥ 20 mmHg at ≥ 1 month (5.1%), mild inflammation (7.6%), and hyphema ≥ 1 mm (2.6%).

Postoperative complications most commonly occur in the first 90 days after surgery and much less frequently beyond 90 days after surgical intervention [31]. According to our study the presence of blood in the anterior chamber was the most common complication, occurring in 55% of cases. Other authors have observed its occurrence in 6.1–85.2% of all surgeries, regardless of whether a single or combined procedure was performed [32–35]. Microhyphema (hyphema) is defined as a small bleed in the anterior chamber composed of liquid blood particles without clot formation. Its height typically ranges from 1 to 2.5 mm. It is caused by hypotonia in the anterior chamber and reflux of blood from the episcleral veins into the Schlemm’s canal, and from there, through the porous trabecular meshwork into the anterior chamber. Reflux of the blood confirms proper tension of the canal walls due to the tensioning effect of the inserted suture and restoration of the physiological AHO. Therefore, this symptom may be treated as an indicator of canaloplasty success, especially because there are no objective methods for measuring the tension of the suture during the surgery. Thus, the absence of microhyphema may be interpreted as inadequate strain of the suture in the canal [33]. This theory is supported by the finding that overall surgical success (IOP <16 mmHg) was achieved in 87% of cases with microhyphema compared to 21% of individuals without microhyphema [32]. The eyes in the latter group required auxiliary goniopuncture more frequently after surgery to achieve the target IOP level. Paradoxically, larger microhyphemas may be absorbed more quickly than are smaller ones, owing to greater trabecular meshwork permeability and better patency of the collector channels [34, 35]. The situation is different in the cases of bleeding during or after trabeculectomy, which occurs at a frequency of 3–43% [36]. This is caused by injury to the iris and trabecular meshwork or blood reflux from the conjunctiva and poses a threat to vision. They may be more profuse, persist for a long time, tend to recur, and cause permanent corneal imbibition [37].

The presence of postoperative IOP spikes after canaloplasty was deliberated by several authors [38–42]. According to Hann et al. [38], the distal part of the conventional outflow pathway accounts for nearly 50% of the AH outflow resistance. The inner wall of Schlemm’s canal, which is adjacent to the AH and anterior chamber, is elastic. The intrascleral placement of the walls of the collector channels causes them to be rigid. Restoring the flow through Schlemm’s canal in the absence of patency in the distal outflow pathways may be a cause of early IOP elevation (≤3 months after surgery). This complication occurs at a frequency ranging from 20 to 35% of cases in the early postoperative period, depending on the study [16–18, 20]. The complication is typically transient in nature and subsides within the first 3 months after surgery. Such an increase may sometimes persist longer (8.3–18.8%), especially in the case of single procedures. It may be caused by irreversible damage to the natural outflow pathway for AH (especially collector channels and intrascleral plexus sclerosis) in advanced glaucoma [24] and is a poor prognostic factor for achieving the desired long-term IOP level after surgery. Other causes of this phenomenon that have been considered include 1) the presence of viscoelasticity in the anterior chamber, 2) postoperative release of inflammatory cytokines that may decrease the outflow through the trabecular meshwork and collector channels [16], and 3) an IOP spike induced by corticosteroids in steroid responders [19]. Anatomical characteristics of trabeculum vary among patients [25] (e.g., a smaller vertical diameter and surface area of Schlemm’s canal, as well as a thinner and narrower trabecular meshwork), which may influence the more frequent occurrence of the complication. In our study, postoperative IOP elevation occurred in 15% of the patients in the early postoperative period, and 5% of them exhibited a persistently high IOP, which has been regarded as postoperative failure. This complication is more likely to occur in eyes with a high baseline IOP [35].

Another important issue is the condition of endothelial cells in patients with glaucoma. At birth, the ECD in a healthy human eye is approximately 6000–7500 cells/mm^2^, with the density decreasing with age by approximately 0.6% per year [43], reaching approximately 2500 cells/mm^2^ at the age of 40 years. In older adults (aged ≥70 years), who are at potential risk of requiring glaucoma surgery, the number of endothelial cells approximately ranges from 1500–2000 cells/mm^2^. In comparison to healthy individuals, patients with glaucoma have a compromised endothelium that corelates with the type, duration, and progression rate of glaucoma, as well as IOP spikes [44]. Specifically, the IOP is recognized as the primary contributor to a compromised endothelium. According to previous studies, patients with POAG had a 3.5–9.2% lower ECD count than that of healthy individuals [45]. Traditional glaucoma filtering surgery is known to affect the endothelium, which may eventually lead to corneal decompensation and vision deterioration. In our study, the rate of ECD loss was stable throughout the follow-up period, with the exception of day 180 postop.

The ab externo canaloplasty procedure is a good therapeutic option for patients with early-to-moderate glaucoma in whom irreversible changes in the AH outflow pathways have not yet occurred. It is important to take into account the potential of AH outflow before surgery because improvement in the AH flow through Schlemm’s canal may not be sufficient in situations where the distal AH outflow pathways are incapacitated. Therefore, this surgical method may be inadequate for patients with a multiyear history of the disease, advanced glaucomatous neuropathy, or a history of IOP exceeding 30 mmHg. Another limitation of canaloplasty is that it is impossible to lower the IOP below the level of pressure in the episcleral veins (8–10 mmHg), which also disqualifies patients with very low target IOP levels.

This study has some limitations. First, it was conducted at a single center and included only patients from Poland. Second, the retrospective nature of the study and exclusion of patients with significantly elevated IOP and advanced glaucoma may have affected the results. Third, there was no control group. The absence of a control group limits our ability to directly attribute the observed outcomes to the surgical intervention. Future studies should consider these limitations. Despite these limitations, we believe that this study may be beneficial since many studies have demonstrated short-term success, research on medium-term outcomes is scarce, and only few data are available regarding long-term results of canaloplasty.

## Conclusions

Canaloplasty may be considered in cases where the target pressure can be achieved with concurrent moderate IOP reduction, owing to the simplicity of the postoperative care and lower frequency of complications. Moreover, it is recognized as a safe procedure with respect to the condition of endothelial cells.

Overall, ab externo canaloplasty is a good option for patients with early to moderately advanced glaucoma and results in a reduction in the IOP by approximately 40% in the Polish Caucasian population compared to pre-surgery levels. When qualifying a patient for surgery, the glaucoma stage, surgical experience of the operator, availability of surgical tools, cost-effectiveness, and the patient’s target IOP must be considered. Further research is required to better understand this surgical procedure and the mechanisms of AH outflow from the eye. Future studies that specifically focus on outcomes in patients with different ethnicities are needed to properly evaluate the efficacy and role of different surgery techniques in glaucoma management.
